# Management of canine wounds using platelet‐rich fibrin (PRF) biomaterial. A case series report

**DOI:** 10.1002/vms3.1236

**Published:** 2024-03-28

**Authors:** Carla S. Soares, Isabel R. Dias, Luís C. Barros, Maria dos Anjos Pires, Pedro P. Carvalho

**Affiliations:** ^1^ Animal and Veterinary Research Centre (CECAV), AL4AnimaLS, Department of Veterinary Sciences, School of Agricultural and Veterinary Sciences University of Trás‐os‐Montes e Alto Douro (UTAD) Vila Real Portugal; ^2^ VetLamaçães, Veterinary Clinic Braga Portugal; ^3^ Center for Investigation Vasco da Gama (CIVG), Department of Veterinary Sciences Escola Universitária Vasco da Gama Coimbra Portugal; ^4^ Vetherapy – Research and Development in Biotechnology Coimbra Portugal

**Keywords:** autologous, dogs, platelet therapy, platelet‐rich fibrin, regeneration, wound healing

## Abstract

**Background:**

The increasing interest in platelet‐based therapies has underwritten the development of novel veterinary regenerative treatments. The haemoderivative platelet‐rich fibrin (PRF) comprises abundant concentrations of platelets and leucocytes, above the physiologic baseline, which are considered essential elements for wound regeneration, stimulating local angiogenesis, cellular migration, proliferation and differentiation, considered essential for skin repair.

**Objectives:**

This study aimed to describe the treatment of eight dogs with naturally occurring cutaneous wounds, where autologous PRF therapy was applied, using a protocol developed by our group.

**Methods:**

Eight dogs, aged between 7‐month and 9‐year old, from different breeds and sexes, were enrolled in this study. Four of these wounds were clinically infected. In three cases, two PRF treatments were performed during the first week of treatment, followed by single weekly treatments from the second week onwards, until exophytic granulation tissues were present. In each case, the treatment was finalized only when complete wound closure was achieved. Wounds did not receive topical antiseptics, antibiotics or topical drugs to promote wound healing during the treatment.

**Results:**

PRF‐grafting treatments were well tolerated in all treated wounds, inducing significant granulation tissue formation. PRF clots acted as a natural tissue filler, promoting epithelization and wound closure, without the requirement of topical antimicrobial/antiseptics application, or additional surgical debridement. Evident skin contraction was recorded in larger injuries and all the treatments resulted in vestigial aesthetic scars where hair growth was also observed.

**Conclusions:**

PRF‐therapy obtained promising results, as an alternative wound treatment, revealing a biological regenerative action, prompting the natural skin healing process.

## Graphical abstract

This study aimed to describe the treatment of eight canine naturally occurring cutaneous wounds, where autologous Platelet‐rich fibrin (PRF) therapy was applied. Eight dogs, aged between 7 months and to 9‐year‐old were enrolled in this study. PRF grafting treatments were well tolerated in all treated wounds, inducing significant granulation tissue formation. PRF clots promoted progressive epithelization and wound closure, even in chronic wounds. Evident skin contraction was recorded in larger injuries and all the treatments resulted in vestigial aesthetic scars.

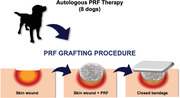

## 1 INTRODUCTION

Recent research supports the therapeutic use of platelet‐rich fibrin (PRF) in human wound treatment, namely chronic and small‐to‐moderate‐sized complex wounds (Miron et al., 2017; Pinto et al., 2018; Shreyas et al., 2019; Vaheb et al., 2020). PRF is defined as a biocompatible and biodegradable natural biomaterial, derived from blood, constituted by a fibrin matrix, containing elevated amounts of platelets and leukocytes, having the capability to release high concentrations of bioactive structural proteins, over time (Ghanaati et al., 2014). The PRF clot contains abundant concentrations of platelets and leucocytes, above the physiologic baseline, equivalent to the cellular elements’ concentration present in the blood volume from which each PRF clot was produced, platelets produced with the centrifugation of blood. Platelets and leukocytes are considered essential elements for wound regeneration, stimulating local angiogenesis, cellular migration, proliferation and differentiation (de Carvalho et al., 2020).

Studies focused on the temporal profile of the PRFs’ secretome have demonstrated that the bioactive proteins are released from human, equine, canine and feline PRF clots, being maintained at the wound bedding even after its application (Ghanaati et al., 2014; Jiménez‐Aristizabal et al., 2017; Kornsuthisopon et al., 2020; Martínez et al., 2015; Soares, Babo, Faria, et al., 2021). Several growth factors (GFs), such as platelet‐derived growth factor‐BB (PDGF‐BB), transforming growth factor β‐1 (TGF‐β1) and vascular endothelial growth factor‐A (VEGF‐A), as well as other important cytokines, are released after platelet and/or leukocytes degranulation, initiated during the centrifugation process (Jiménez‐Aristizabal et al., 2017; Pavlovic et al., 2021). A recent in vitro study demonstrated the active release of PDGF‐BB, TGF‐β1 and VEGF‐A from canine PRF until day 10 after its production and verified an initial burst release of interleukin‐8 after 1 day of PRF preparation (Soares, Babo, Faria, et al., 2021). The PRF's success in the human clinical context has been supported by both in vitro and in vivo scientific research (Ghanaati et al., 2014; Masuki et al., 2016; Naik et al., 2013; Preeja & Arun, 2014). Additionally, the increasing interest in human platelet–based therapies has also contributed to the development of novel veterinary treatments, but a lack of information exists in the veterinary clinical field regarding PRF therapy, especially in wound regeneration, clinical scenarios already acceptable in human patients (Soares et al., 2018; Soares, Dias, et al., 2021).

This article documents the treatment of eight naturally occurring wounds from different aetiologies in dogs, where autologous PRF therapy was applied. The PRFs were produced using a protocol developed by the group (Soares, Babo, Faria, et al., 2021; Soares, Dias, et al., 2021). The clots were applied directly into the wounds as a biological patch. The present work also documents the follow‐up evaluation of the treated wounds.

## 2 MATERIALS AND METHODS

### 2.1 Canine population and production of autologous PRF clots

This study was approved by the Ethics Committee of the Ethics Committee of the University of Trás‐os‐Montes E Alto Douro (Doc03‐CE‐UTAD‐2020). The owners of the animals involved in the study signed a written informed consent.

Eight domestic client‐owned dogs (five females and three males), aged between 7‐month and 9‐year old, with different aetiologies, were referenced to be enrolled in this study (Table 1).

**TABLE 1:** Demographic and clinical characterization of the eight dogs treated with platelet‐rich fibrin (PRF) therapy enrolled in the study.

Case	Age	Sex	Weight (kg)	Breed	Origin of the lesion and localization	Characteristics of the lesion
1	4 ye	Female, intact	13	Cocker Spaniel	Dog bite, left cervical region	Infected wound (6 h, according to owner)
2	8 ye	Female, spayed	40	German Shepherd	Subcutaneous ruptured abscess, right scapular region	Necrotic tissue infected wound (>1 day)
3	7 ye	Male, neutered	32	Retriever Labrador	Traumatic wound, posterior limb foot pads	Chronic wound (>1 month, sutured previously, but recurred)
4	9 ye	Male, intact	30	Crossbreed German Shepherd	Ulcerative lesion due to leishmaniosis (confirmed 1 week after), right thoracic carpus	Infected wound (1 week)
5	5 ye	Female, spayed	7	Crossbreed	Dehiscence of surgical repair of a dog‐bite lesion, left cervical region	Infected wound (3–4 days)
6	9 ye	Male, neutered	28	American Pit Bull Terrier	Surgical wound dehiscence due to radical surgical tumour resection, left metacarpus	Accentuated tissue loss and ligament exposition (2 days)
7	8 ye	Female, spayed	38	Crossbreed Portuguese Serra‐da‐Estrela	Myiasis (maggot larvae of flies) infestation, right posterior ear pinna	Accentuated tissue loss cartilage exposition week
8	7 mo	Female, intact	13	Border Collie	Laceration in the interdigital region, II–III digits, right thoracic foot pads	Chronic wound (with 24 days), suffering dehiscence after 2 suturing procedures, and 1 surgical tissue adhesive application

Abbreviations: mo, months; ye, years.

The animals were first assisted by a general veterinary clinician and were later referred to a specialized regenerative wound therapy clinician. The animals presented lesions from different aetiologies. Four wounds were identified as infected (cases 1, 2, 4 and 5).

### 2.2 Protocol for the production of the PRFs

Each PRF clot was produced using an aseptic technique as previously described (Soares et al., 2018; Soares, Babo, Faria, et al., 2021; Soares, Dias, et al., 2021). Briefly, 5 mL of venous blood was collected to sterilize conical base dry polypropylene tubes and to produce one PRF clot. The centrifugation at 580*g* (3000 rpm) was immediately performed after the blood collection, for 10 min, at room temperature. It used an in‐clinics 45° angle rotor centrifuge (Ortoalresa, RT 114, NS 080214/02, *ø* 8.2 cm). The blood was left to rest inside the tube for a maximum time of 60 min after the centrifugation step, for the cases in which PRF polymerization did not occur during or immediately after the centrifugation step. The PRF clots were harvested from the tubes, and the red fraction was removed using sterile tweezers and scissors.

### 2.3 PRF protocol: grafting procedure and treatment

On the first day of treatment (D1), all the wounds were scored from 1 to 4 according to their cleanliness and condition score (1: clean wound; 2: clean‐contaminated wound; 3: contaminated wound, without necrosis, or the presence of necrotic tissue without infection; 4: dirty‐infected wound, necrosis and pus present, traumatic wounds greater than 4‐h old) (Herman & Bordoni, 2020). Thereafter, the skin wounds were mechanically debrided, and necrotic tissue was removed, if present. Thereafter, before each new treatment, all the lesions were irrigated with a sterile saline solution to remove all inflammatory exudate or debris.

The number of PRFs applied depended on each lesion size, considering that 1 PRF was applied for each 1.5–2.5 cm^2^ of the wound area, and a maximum of 4 PRFs/wound were applied.

Most of the patients received conventional systemic medicines, implemented by the first assistant veterinary clinician (Table 2). In three cases, two PRF treatments were performed during the first week of treatment, and single treatments were applied from the second week until wound epithelization was reached (cases 2,3 and 5). In the other five cases, two PRF treatments that were performed during the first and second week of treatment, followed by single treatments were applied from the second week until wound epithelization was reached. The application of PRFs was suspended once proliferative and exophytic granulation tissue was observed in the wound bedding. Continuity of the bandaging procedure was performed equally until complete wound closure was reached.

**TABLE 2:** Therapeutic schedule for each of the eight treated dogs, during the platelet‐rich fibrins’ (PRFs’) treatment period.

Wound case	Pharmacological systemic treatment instituted
1	Meloxicam (Meloxidyl, Ceva) 0.1 mg/kg *q* 24 h, during 5 consecutive days
	Amoxicillin–clavulanic acid (Clavaseptin, Vetoquinol) 20 mg/kg *q* 12 h, for 8 days
2	Meloxicam (Meloxidyl, Ceva) 0.1 mg/kg *q* 24 h, during 5 consecutive days
	Amoxicillin–clavulanic acid (Clavaseptin, Vetoquinol) 20 mg/kg *q* 12 h, for 10 days
	Metronidazole (Metrobactin, Dechra) 15 mg/kg *q* 12 h, for 10 days
	Tramadol (Tralieve, Dechra)3 mg/kg *q* 12 h, for 5 days
3	Both started to be administrated 1 week before PRF therapy
	Meloxicam (Meloxidyl, Ceva) 0.1 mg/kg *q* 24 h, during 5 consecutive days
	Amoxicillin–clavulanic acid (Clavaseptin, Vetoquinol) 20 mg/kg *q* 12 h, for 10 days
4	Meloxicam (Meloxidyl, Ceva) 0.1 mg/kg *q* 24 h, during 5 consecutive days
	Amoxicillin–clavulanic acid (Clavaseptin, Vetoquinol) 20 mg/kg *q* 12 h, for 10 days
5	Meloxicam (Meloxidyl, Ceva) 0.1 mg/kg *q* 24 h, during 5 consecutive days
	Cephalexin (Cefabactin, Dechra) 20 mg/kg *q* 12 h, during 10 consecutive days
	Buprenorphine (Bupredine, Dechra) 0.02 mg/kg *q* 12 h, during the initial 24 h, followed by tramadol (Tralieve, Dechra) 3 mg/kg *q* 12 h, for 5 days
6	Meloxicam (Meloxidyl, Ceva) 0.1 mg/kg *q* 24 h, during 5 consecutive days
	Amoxicillin–clavulanic acid (Clavaseptin, Vetoquinol) 20 mg/kg *q* 12 h, for 10 days
	Buprenorphine (Bupredine, Dechra) 0.02 mg/kg *q* 12 h, during the initial 2 days
7	Meloxicam (Meloxidyl, Ceva) 0.1 mg/kg *q* 24 h, during 5 consecutive days
	Amoxicillin–clavulanic acid (Clavaseptin, Vetoquinol) 20 mg/kg *q* 12 h, for 10 days
	After D24: auricular suspension containing Miconazole 23 mg + Prednisolone 5 mg + Polymyxin B 5500 IU (Conofite, Ecuphar), 5 drops/right ear, 14 days
8	Not administrated during PRF therapy (administrated before, but with no ATB or NSAID in the previous 15 days)

Abbreviations: ATB, antibiotics; D, day; mg, milligrams; NSAID, non‐steroid anti‐inflammatory drug; *q*, every *n* hour.

### 2.4 Wound area evaluation and statistical analysis

The wound area was documented and assessed at each time point. The wound depth was not considered for evaluation. Most of the patients were also re‐evaluated at a minimum of 2 months after wound closure.

The wound area during the healing progress was calculated by the same researcher using ImageJ software (version ImageJ: 2.1.0/1.53c). A Spearman's rank correlation coefficient (*rs*) was applied to determine the association among the initial wound area, the wound healing duration, the number of PRF treatments performed, the number of PRFs applied at the first treatment and the total number of PRFs applied and the wound contamination score. The percentage of wound contraction was calculated using the following formula: [(wound area at day 1 − wound area at specific time point)/wound area at day 1] × 100 (Soares, Dias, et al., 2021). Results were expressed as the median, and interquartile ranges (IQR). Statistical analysis was conducted using Prism (Version 6 GraphPad Software Inc.). Statistical significance was considered *p* < 0.05.

## 3 RESULTS

### 3.1 Macroscopic analysis of the wounds

**FIGURE 1:**
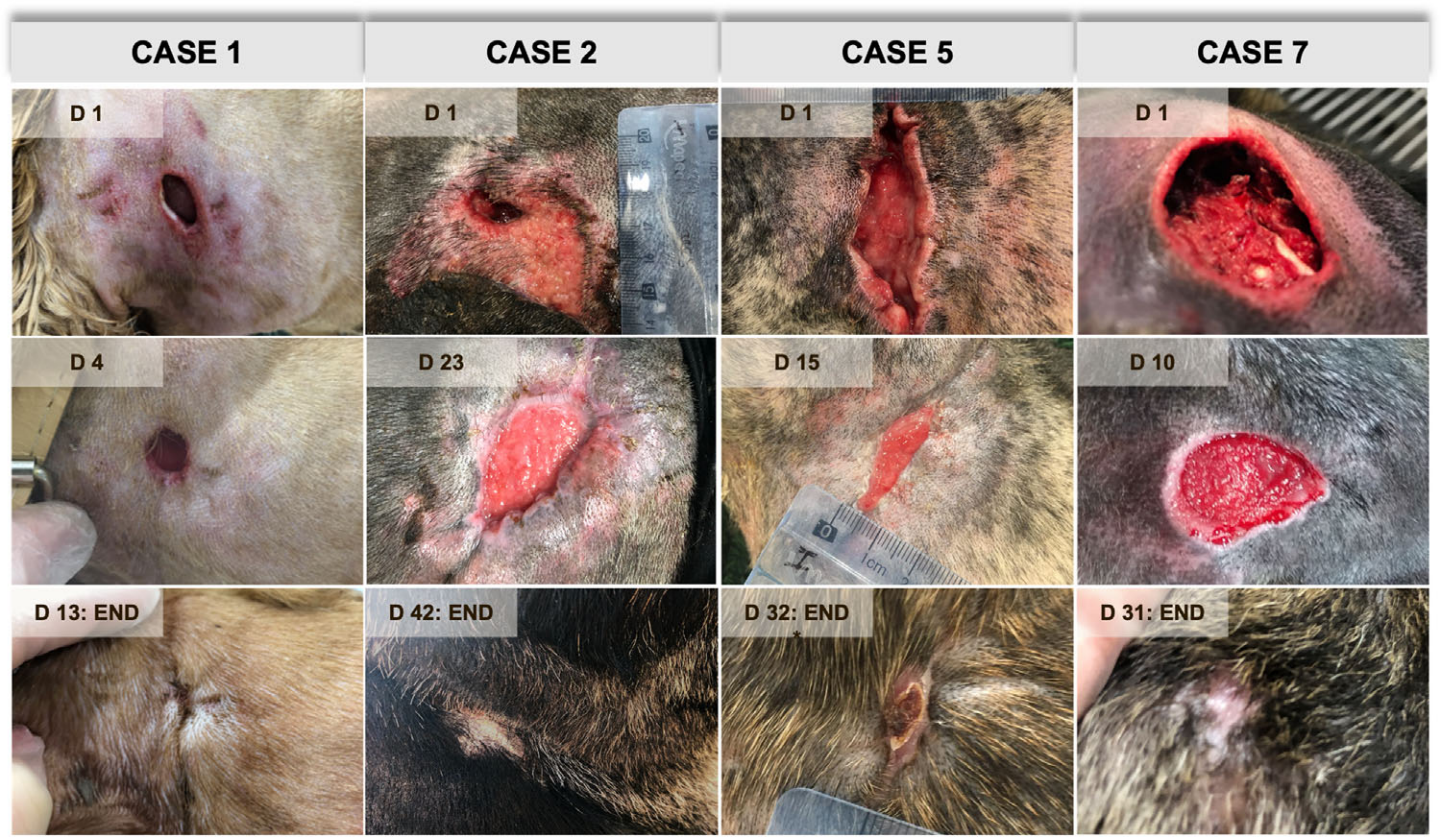
Macroscopic representation of four of the eight canine chronic wounds treated with platelet‐rich fibrin. Wounds from cases 1, 2 and 5 were initially infected, presenting purulent exudate. D, day; END, finalization of the study/complete wound closure.

**FIGURE 2:**
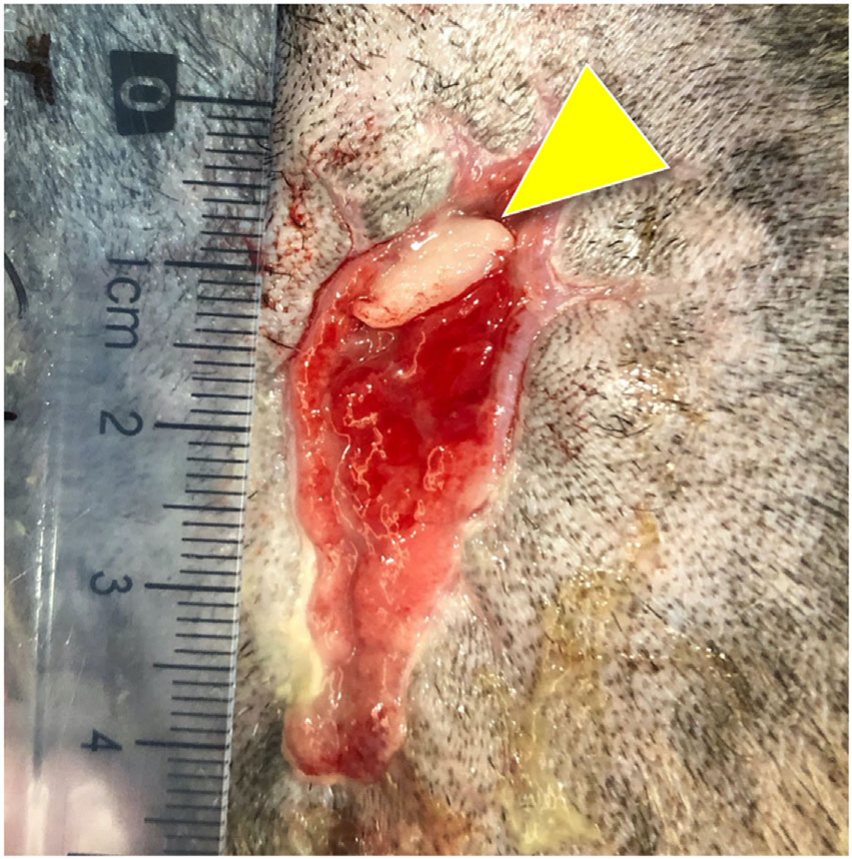
The appearance of the wound in case 5, after three autologous platelet‐rich fibrin (PRF) grafting procedures. In this case, the clot previously applied was still visible (yellow arrowhead) in this treatment, integrated into the new formerly granulation tissue.

All PRF treatments were well tolerated, and allergic manifestation, such as skin rash, pruritus or even fever, was not reported (Figure 1). All the PRF‐grafting procedures induced a noteworthy granulation tissue formation, a highly friable and intense‐reddish material at the wound bedding. In most cases, the mainstream PRF clots applied were not visible in subsequent treatments, although they could be found in occasional cases attached and integrated within the recent granulation tissue (Figure 2).

In case 3, the epithelization process was more evident than granulation tissues formation after PRF therapy. This wound, located in the hind limb, was considered closed at 28 days, despite being considered to have deficient epithelization. Nevertheless, 3 weeks after it was open, a digit amputation was performed as a squamous cell carcinoma lesion was diagnosed in the excised tissue.

The molecular analysis by polymerase chain reaction of the animal in case 4 revealed the infection by *Leishmania* sp. This patient did not complete the study, and humanized euthanasia was performed, as requested by the owners once they could not treat the animal due to financial concerns. Nevertheless, the wound of this canine received two PRF treatments, and in 2 weeks, the wound was reduced by 42.92%.

Four of the assessed wounds were infected on day 1, containing purulent exudate. Case 1, resulted from a dog bite injury, and according to the owner, it would have around 6 h. Nevertheless, the characteristics revealed by the medical examination of the injury, namely the presence of purulent yellow materials with odour, and the inflammatory satellite dermatitis in the surrounding tissue (Figure 1, case 1/day 1) suggested that the injury had not an acute onset.

In case 7, external fungal otitis (*Malassezia* sp.) possibly delayed the wound healing process, as the owner reported the dog scratched the affected ear. Additionally, the interior facet of the pinna was also injured. At this time and considering the absence of a severely exudative ear canal, the affected ear channel was cleaned with a lipolytic and keratolytic veterinary solution, and the dog started topical auricular treatment.

The presence of exudate was observed in the larger and deeper wounds, at the initial time points of the grafting treatments. Nevertheless, no infection signs were observed in the peripheric area and/or in the wound bedding during the PRF treatments. Ischaemic or necrotic tissue was not observed in any of the cases, under any circumstance. The complete wound closure (*n* = 7) occurred in a medium period of 31 days (13–42 days), following an estimated reduction rate of 0.23 cm^2^/day.

Wound contraction, re‐epithelialization and crust formation were progressively perceived, in all the cases. Most of the cases resulted in dry crust formations, except for case 7, where a serous crust was observed after day 18.

**FIGURE 3:**
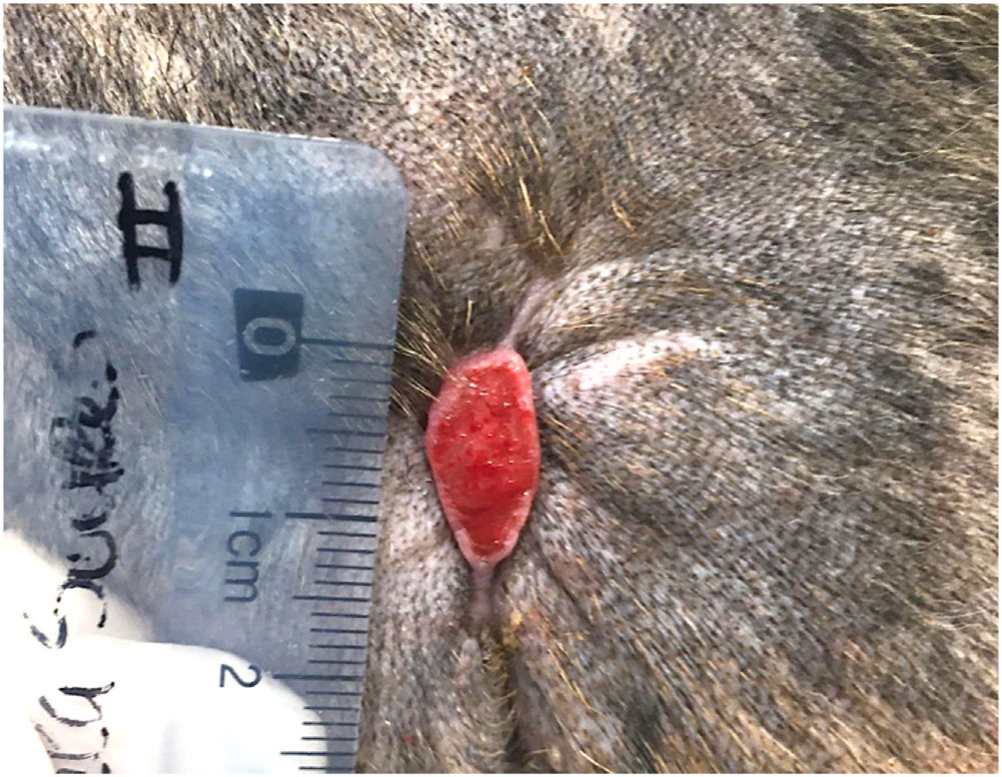
Features of considerable wound contraction were observed in case 5, on day 26, with epithelization formation.

All the wounds recorded a notorious centripetal wound healing, from the periphery to the centre of the lesion. Moreover, all the treatments resulted in vestigial aesthetic scars, and skin contraction was recorded in larger injuries (Figure 3). Hair growth was also observed. Two wounds relapsed after PRF therapy (cases 2 and 3). All the other dogs recorded no wound recurrence for a follow‐up period of 2–12 months.

### 3.2 Assessment of the wound area along the time

**FIGURE 4:**
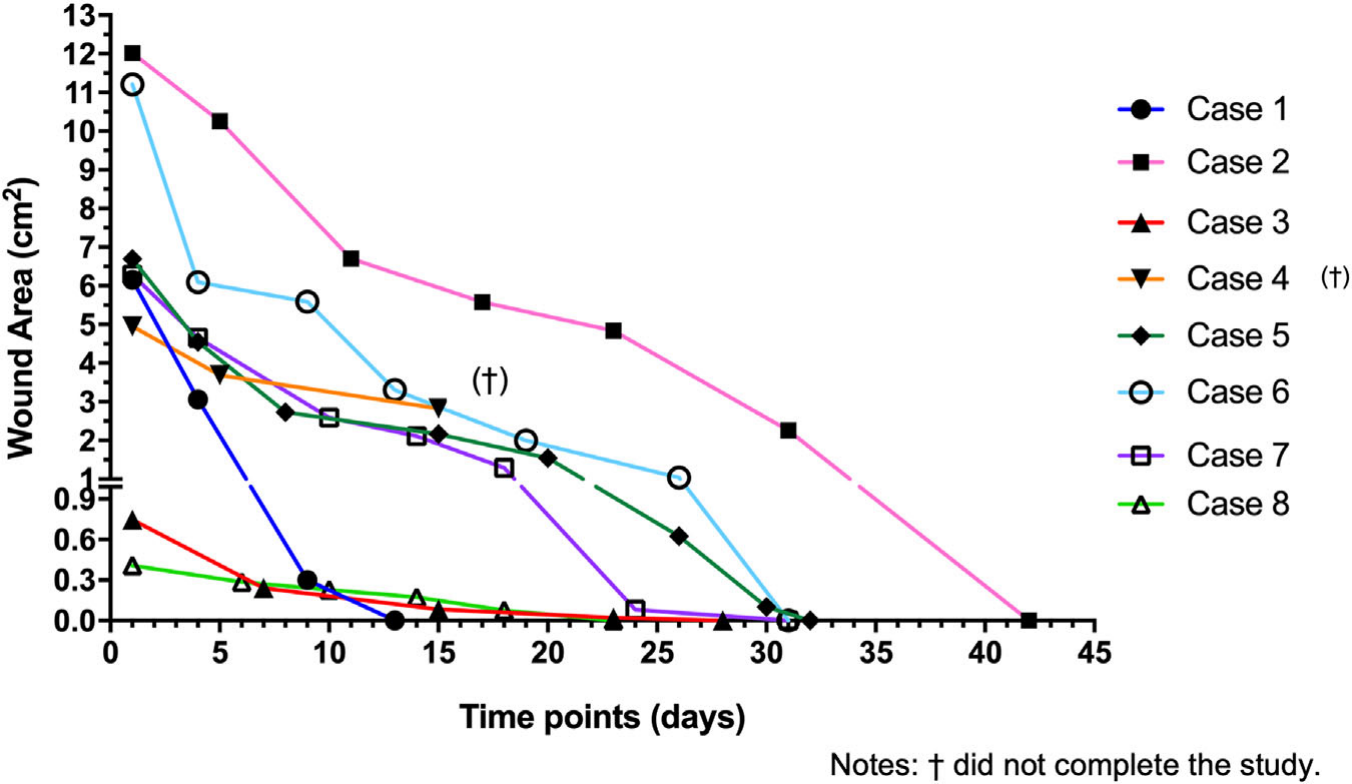
Quantitative assessment of wound area of the eight wounds treated with autologous platelet‐rich fibrin from the initial appointment to the end of the study.

The wound area of each skin wound documented throughout time is characterized in Table 3. The wound area recorded for each lesion is expressed in Figure 4. In all wounds, a consistent and significant contraction of the skin was observed within the initial 2 weeks of PRF therapy, revealed by a slope in the wound contraction percentage curve plot, for each treated wound.

**TABLE 3:** Resumed characterization of the eight wounds treated by platelet‐rich fibrin (PRF)‐therapy, and events documented in the study (considering D1 as the baseline).

Case	Initial wound area (cm^2^)	Wound healing duration (days)	Number of PRF treatments	Total number of wound treatments	Total number of PRF clots applied	Score of cleanliness/contamination degree of wounds (Herman & Bordoni, 2020)	Ongoing therapeutic drugs	Follow‐up period
1	6.15	13	3	4	10	4	ATB NSAID	After 2 months: no wound recurrence; skin with normal appearance
2	12.02	42	5	7	17	4	ATB NSAID ANALG	A nodule (±1 cm) was identified dorsally to the wound, immediately before PRF therapy After 10 months: wound recurrence (small area), associated with nodule growth (2.3 cm diameter) Owners accepted the surgical removal of the nodule (histopathology revealed extraskeletal chondrosarcoma)
3	0.75	28	3	5	2	3	ATB (Started to be administrated 1 week before PRF therapy)	After 3 weeks: wound recurrence; surgical amputation of the digit was performed, and histopathology revealed a dermic squamous cell carcinoma, not detected by appositional cytology performed at the initial consult presentation
4	4.95	15	2	3	4	4	ATB NSAID	This animal did not complete the study, being euthanized according to the owner decision or because of disease after the confirmation of *Leishmania* sp. infection
5	6.70	32	5	8	12	4	ATB NSAID ANALG	After 6 months: no wound recurrence; skin with normal appearance
6	11.21	31 days	5	7	10	3	ATB NSAID ANALG	After 12 months: no wound recurrence; skin with normal appearance
7	6.29	31	4	7	7	3	ATB NSAID TOP‐ANTIFUNG (>D24) Not observed	After 12 months: no wound recurrence; skin with normal appearance
8	0.41	23 days	2	6	2	2	Not administrated After D14: an ointment containing vitamin A + zinc applied only in the interdigital plantar region	After 2 months: no wound recurrence; skin with normal appearance

Abbreviations: ANALG, analgesic drug; ATB, antibiotic drug; D, day; NSAID, non‐steroidal anti‐inflammatory drugs; TOP‐ANTIFUNG, topical antifungal drug.

The initial surface area of the eight treated wounds ranged from 0.41 to 12.02 cm^2^, with a median surface area of 6.22 cm^2^ (IQR 1.80 to 6.22 cm^2^), at day 1, immediately before the first PRF treatment.

A progressive median wound contraction percentage along the PRF therapy was calculated in the seven cases enrolled in the study: 30.88% (IQR 25.675%–49.11%) at the end of the first week of treatment (until day 7), increasing to 62.76% (IQR 47.48%–84.18%) at the second week (until days 14–15), with one lesion achieving the complete closure (case 1). Also, at this time point, case 4 did not proceed any further, being excluded from the study (as previously stated).

In the third week (from 15 to 21 days), six cases were ongoing, recording a median wound contraction of 79.41% (IQR 65.22%–81.63%). In the fourth week of treatment (days 22–28), from the six cases being assessed, two achieved complete healing, with a median wound contraction of 94.74% (IQR 82.93%–100%). In the fifth week of treatment (days 29–35), three of the four ongoing cases concluded the study (Figure 1, case 5/day 32 and case 7/day 31). The only wound still not completely healed in the study, at this time, was the larger lesion which achieved complete resolution on day 42.

A statistically significant positive correlation was found between the initial wound area and the wound healing duration (*rs* = 0.8289, *p* = 0.0278), and between the initial wound area and the number of PRF treatments performed (*rs* = 0.9543, *p* = 0.0048). However, no correlation was found between the duration of the wound healing process and the number of PRF clots applied at the first treatment, or the total number of PRF clots applied during the complete treatment (*rs* = 0.3056, *p* = 0.5000 and *rs* = 0.7000, *p* = 0.0857, respectively). A statistically significant correlation was found between the total number of PRF clots applied and the score of cleanliness and condition of the wounds (*rs* = 0.8758, *p* = 0.0214).

## 4 DISCUSSION

PRF rational use stems from the fact that the clot acts as a natural tissue filler, being an important trigger for the local healing progress, promoting both neoangiogenesis and tissue remodelling, by releasing high concentrations of bioactive structural proteins (Ghanaati et al., 2014). The concentration of leukocytes observed within the PRF also directly guarantees tissue regeneration (Ozer & Colak, 2019).

Overall, a progressive wound contraction was documented in the cases reported herein, being more evident within the initial 2 weeks of PRF therapy: The second PRF‐grafting procedure was executed in a median of 4.5 days after the initial wound presentation, associated with a wound contraction of 30.88%, at the first week. A median wound contraction of 62.76% was achieved between days 8 and 15 after two PRF‐grafting treatments (*n* = 8), where case 1 accomplished complete wound closure. This issue suggests a sustained healing pattern provided by a local delivery of high concentrations of both GFs and cytokines released from the PRF matrix, as described in the literature (Soares et al., 2020; Soares, Babo, Reis, et al., 2021). A statistically positive correlation was found among the initial wound area, the wound healing duration and the number of PRF treatments performed. Therefore, larger wounds require a higher number of PRFs and a longer time to heal. Nevertheless, the duration of the wound healing process is not associated with the number of PRF clots applied at the first treatment, or with the total number of PRF clots applied during the complete treatment. Contaminated wounds or wounds with a higher score required a higher number of PRF clots applied. In all the cases, PRF clots were perfectly integrated into the wound site, being macroscopically degraded in situ, with no rejection or other adverse reaction, or necrosis.

From the fewer in vitro studies on canine PRFs, some have demonstrated PRF ability in reducing the expression of inflammatory cytokines such as TNF‐α and IL‐β1, stimulating the expression of collagen production‐associated genes and some GFs (PDGF‐B, TGF‐β1 and VEGF‐A) (Kornsuthisopon et al., 2020; Tambella et al., 2020). The GFs released are recognized as endogenous peptides that regulate both fibroblast and peripheral stem cell migration, proliferation and differentiation, also promoting angiogenesis, which is crucial for wound healing processes (Ehrenfest et al., 2010; Toffler et al., 2009). Moreover, progenitor stem cells were detected in platelet concentrates, the majority from the hematopoietic lineage, having the capacity to promote the maturation of endothelial cells and local neoangiogenesis; stem cells from nonhaematopoietic lineages were also found, having the ability to differentiate into mesenchymal cells (e.g. osteoblasts and chondrocytes) (Bielecki & Ehrenfest, 2012).

The number of clinic visits for wound treatment and bandage change was reduced using this PRF therapy when compared to the bandage changes/wound treatment necessary on conventional medical wound bandaging/treatments that are required for each 2–3 days until wound closure is achieved (Tobias, 2012). The cases presenting extended or profound injuries (1, 6, 7 and 8), and one case with moistening crusted lesions due to its localization (case 8, lesion in the interdigital location) required additional regenerative biomaterial in the second week.

Only two of the seven that concluded the study experienced wound relapse, and latter histopathological examination exposed the presence of neoplastic lesions, probably present before the PRF therapy (extraskeletal chondrosarcoma associated with a nodule in case 2, and a dermic squamous cell carcinoma associated with a chronic wound that did not respond to surgical intervention, in case 3). In case 2, the previous imprint cytologic exam was negative for neoplastic cells. Recent works have studied platelet‐derived formulations, such as platelet‐rich plasma, as a co‐adjuvant therapy in cancer treatment, helping the slower growth of the tumour (Barbieri et al., 2019; Luzo et al., 2020). Nonetheless, the influence of PRF on the development of neoplasia has never been clearly stated by human clinical researchers, and it is important to note that being a recent methodology, there are still many unexplored features (Gupta et al., 2011; Ozer & Colak, 2019; Steenvoorde et al., 2008).

Canine patients involved in the present research tolerated extremely well the repeated wound manipulation, involving the PRF‐grafting and the associated consecutive dressings, without the use of topical anaesthesia. Analgesic action associated with PRF therapy has been reinforced by recent reports (Albilia et al., 2020; Al‐Hamed et al., 2017; Soares, Dias, et al., 2021).

PRF therapy has been claimed to have effective topical antimicrobial activity, and also it was confirmed by our group (data not shown) (Burnouf et al., 2013; Feng et al., 2020; Shariati et al., 2020). The inexistence of wound infection in this research may uncover an important intrinsic antimicrobial property of PRF clots, especially considering that no local antiseptic was applied and that four wounds were unequivocally infected. Case 8 was treated only with PRF therapy, and case 3 had antibiotic administration only in the first 3 days of PRF treatment. This study found a statistically significant correlation between the score of cleanliness/contamination of wounds and the total number of PRFs applied.

Technically, PRF methodology was revealed to be an easy and cost‐effective therapy, with a simple obtention protocol and with low variability in clot formation between individuals. Furthermore, the PRF therapy technique can be pointed as an environmentally sustainable technique, as polypropylene tubes used to produce the standardized PRFs could be reused, envisioning waste reduction and supporting ecological practices in clinical research. The tubes were used after both appropriate decontamination and washing, followed by autoclave sterilization, being this plastic polymer cost‐efficiently produced, thermoresistant and considered to have low cell attachment (Maddah, 2016; Soares, Babo, Faria, et al., 2021; Sun et al., 2019).

A peripheral‐to‐the‐centre healing pattern was observed in all wounds. Although normal (due to epithelial cell proliferation and migration towards the centre of the wound during the wound closure), it is important to note that a significant burst of granulation tissue was documented, above the physiologic skin regeneration observed in medical wound treatments, commonly designated as conventional secondary intention healing.

The cell proliferation stage of soft tissue healing begins 2–3 days after the skin injury, with the beginning of the formation of granulation tissue (Park et al., 2017). The granulation tissue results from the cell migration promoted by the presence of PDGF and TGF‐TGF‐β1, released by both platelets and inflammatory cells, previously recruited for the region, during the parallel haemostasis‐inflammation stages. Fibroblasts, cells responsible for matrix synthesis, myofibroblasts, which are responsible for contracting the lesion throughout the regenerative process, and endothelial cells, inducers of the neoangiogenesis process, are stimulated by the peptides VEGF and fibroblastic GF and constitute the granulation tissue (Park et al., 2017; Tottoli et al., 2020). The re‐epithelialization occurs later, being marked by the migration and proliferation of keratinocytes that are found on the periphery of the lesion, towards the centre of the wound, while the granulation tissue develops to fill and approximate the edges of the defect. The last phase of wound repair is the remodelling phase, more prolonged, in which active cells undergo apoptosis, and an intense deposition of extracellular matrix, composed of fibronectin, glycosaminoglycans, proteoglycans and hyaluronic acid, and the type III collagen the most predominant, which is being gradually replaced by type I collagen (Tottoli et al., 2020). Moreover, the authors trust PRF clots constitute a temporal‐releasing patch of essential GFs, released in increased concentrations, mandatory for all the stages of wound repair, and thenceforth, mimicking the physiologic process. The burst of GFs is subsequently provided by each PRF‐grafting treatment, according to the protocol established, consisting of two PRF treatments during the first 1–2 weeks of treatment (the critic repair stage), replaced then by single treatments, until epithelization is notably observed.

The authors consider that more studies are required to access the PRF clinical performance, and outcomes. The present study assessed the evolution of the wound area in the presented cases, using image software. Nevertheless, wound depth is an important feature that should be addressed, especially when a study is targeting wound healing assessment using a different treatment approach, such as PRF therapy. The authors would like to state that most of the cases documented here were already receiving systemic antimicrobials, prescribed by the first general veterinary clinician.

## 5 CONCLUSION

The preliminary features frame PRF therapy as a promising alternative medical wound therapy, with biological and regenerative interest for veterinary patients, especially those suffering from co‐mobilities or medical issues that enable surgical wound interventions. Owing to its stimulatory effect on angiogenesis and epithelialization, PRF behaviours as an excellent biomaterial act as a temporal‐release patch. PRF discharges bioactive peptides in situ that endorse the physiologic restoration of tissue integrity. Additionally, PRF application may reduce the use of additional antimicrobial/chemical agents generally used in wound management, unveiling a possible antimicrobial local effect, and an environmental protective medical technique under the *One Health* context. PRF therapy constitutes a biologically cost‐effective strategy, reducing the need for surgical reconstructive interventions, occasionally restricted by the existence of possible patient morbidities, possible clinical post‐operative complications, or even by economic restraints. Additionally, the number of clinical visits for bandage change was reduced by applying PRF therapy compared with conventional medical wound treatments, reducing clinical residues associated with wound treatment.

## AUTHOR CONTRIBUTION

*Study design*: Carla S. Soares, Maria dos Anjos Pires and Pedro P. Carvalho. *Data acquisition and investigation*: Carla S. Soares, Isabel R. Dias, Luís Barros and Maria dos Anjos Pires. *Software*: Carla S. Soares and Maria dos Anjos Pires. *Statistical analysis*: Carla S. Soares and Maria dos Anjos Pires. *Resources*: Carla S. Soares, Luís Barros and Maria dos Anjos Pires. *Writing – first draft*: Carla S. Soares, Isabel R. Dias and Maria dos Anjos Pires. *Writing – review and editing*: Carla S. Soares, Isabel R. Dias, Maria dos Anjos Pires and Pedro P. Carvalho. *Final approval of the version to be published*: Carla S. Soares, Luís Barros, Isabel R. Dias, Maria dos Anjos Pires and Pedro P. Carvalho. *Project administration and supervision*: Maria dos Anjos Pires and Pedro P. Carvalho.

## CONFLICT OF INTEREST STATEMENT

Pedro P. Carvalho is the C.E.O. and Founder of Vetherapy, a biotechnology company that commercializes cellular therapies for veterinary medicine. All the other authors declare that there is no conflict of interest regarding the publication of this article.

### PEER REVIEW

The peer review history for this article is available at https://publons.com/publon/10.1002/vms3.1236.

## ETHICS STATEMENT

This study was approved by the Ethics Committee of the University of Trás‐os‐Montes e Alto Douro (Doc03‐CE‐UTAD‐2020). The owners of the animals involved in the study signed a written informed consent.

## Data Availability

Data sharing is not applicable to this article as no new data were created or analysed in this study.
